# Comparison of the Effect of Hydroalcholic Extract of *Alhagi maurorum* and Hydrochlorothiazide on Excretion of 4–10 mm Kidney and Ureteral Stones in Adults: A Randomized Prospective Study

**DOI:** 10.1155/2023/6624981

**Published:** 2023-08-14

**Authors:** Sadrollah Mehrabi, Parisa Beigi, Zeinab Salehpour

**Affiliations:** ^1^Clinical Research Development Unit, Shahid Beheshti Hospital, Yasuj University of Medical Sciences, Yasuj, Iran; ^2^Medicinal Plants Research Center, Yasuj University of Medical Sciences, Yasuj, Iran

## Abstract

**Objective:**

The prevalence of kidney stones and their complications is high. The review of the literature showed the therapeutic effects of *Alhagi maurorum* extract on urinary tract stones. This study reviewed the *Alhagi* plant's hydroalcholic extract's effect on eliminating kidney and ureteral stones compared to hydrochlorothiazide.

**Materials and Methods:**

In this randomized prospective study, from March 2019 to September 2021, 80 patients over 18 years of age with kidney stones in the upper ureter with a size of 4–10 mm were divided into two groups based on the block random allocation method. The first group received hydrochlorothiazide tablets (50 mg), and the second group received 1 gram/day of the hydroalcholic areal extract of *Alhagi maurorum* in a two-divided capsule. The mean size and number of stones, renal function tests, and side effects were checked and compared in both groups before and after the study.

**Results:**

Mean age, sex, serum urea level (*P*=0.351), serum creatinine (*P*=0.393), stone size (*P*=0178), and the number of stones (*P*=0.052) before intervention were similar. After intervention, the size and number of stones diminished, up to 70% in both groups. However, there was not a statistically significant difference between the two groups.

**Conclusion:**

The study showed that *Alhagi maurorum* is as effective as hydrochlorothiazide in treatment of kidney and ureteral stones with no significant complications and is promising.

## 1. Introduction

The risk of developing kidney stones during life is about 5–10% and often occurs in both sexes in the early 20–40 years. Most people with kidney stones suffer from acute colicky pain that interferes with daily activities and may require narcotic analgesics to relieve pain. Kidney stones can also cause urinary tract infections, obstruction, hydronephrosis, hematuria, and pyuria [1–3]. Several synthetic and herbal medicines have been used to treat kidney stones and stone excretion, which have comparable results and are often effective in stones with a size of less than 6 mm [4, 5].

*Alhagi maurorum* or camelthorn is one of the ancient plants that have 3–5 species [6]. Its shrubs are semishrubby and semiwoody, reaching a height of 50–150 centimeters. It has green stems with sharp yellow spines. The seeds are placed next to each other in a reddish-brown shell bound between the seeds. New shoots can grow up to 20 feet in height from the mother plant [3, 7, 8]. It is used to treat various diseases such as gastroenteritis, ulcers, fever, inflammation, angina, migraine headache, toothache, liver disorders, kidney and urinary tract infections, and hypertension. It has an antispasmodic and antioxidant property [6, 9].

Kidney stones and their complications are common. Conventional treatments are expensive, and there is not any standard drug with good efficacy, so it will be necessary to find new and effective remedies [4, 5, 7]. There is some evidence in the medical literature that shows therapeutic indication of *Alhagi maurorum* on both prevention and treatment of renal stones in animal studies, although in some of them it had only diuretic effects, while in others, it had a spasmolytic effect on the smooth muscle and helped passage of ureteral stones [7, 8]. Due to controversy about preventive and therapeutic effects of *Alhagi maurorum* on urinary stones and absence of a standard drug for passage of stones and well-known diuretic and spasmolytic effects of the *Alhagi* extract, we decided to study the efficacy of the hydroalcholic extract of *Alhagi* on excretion of the urinary tract's stones in comparison with oral hydrochlorothiazide.

## 2. Materials and Methods

### 2.1. Study Design

In this randomized clinical trial, from March 2019 to September 2021, eighty patients (40 using 50 mg of the hydrochlorothiazide tablet and 40 using the *Alhagi mauroru*m areal extract) over 18 years of age with kidney and upper ureteral stones with a size of 4–10 mm were referred to the urology clinic after obtaining approval from the ethics committee. In addition, before any intervention, a written informed consent form was signed by all participants.

**Figure 1:**
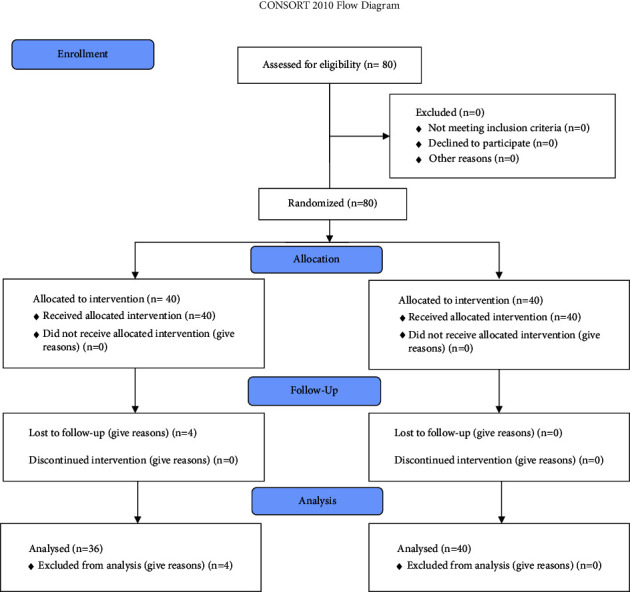
Consort 2010 flow diagram of the study.

After preparation of the combined extract, the target population was first examined for urinary tract infections, azotemia, coagulation disorders, pregnancy, hypertension, severe cardiopulmonary disease, and herbal or drug history of allergies. They were assigned to one of the two groups by the randomly assigned block allocation method with quadruple blocks. All patients had a complete history of physical examination, baseline serum tests (CBC, Na, K, PT, and PTT), kidney function tests (BUN and Cr), and complete urine tests and a urine culture. They were told to drink 10–12 glasses of water daily and exercise and walk for at least 30 minutes a day. In the first group, hydrochlorothiazide tablets, one 50 mg tablet, were given every night for two weeks, and in the second group, 1 gram of the areal hydroalcholic extract of *Alhagi mauroru*m per day (equivalent to two capsules) was given separately every 12 hours with a glass of water for two weeks. In both the groups, oral diclofenac sodium analgesia was used according to weight to relieve pain. If there was no response to analgesia or intervention indications, necessary lithotripsy treatment was performed. Two weeks later, the patients were visited again, and the results of follow-up tests and ultrasound were recorded and analyzed (Figure 1).

### 2.2. *Alhagi maurorum* Usage

After collecting the plant from Dena Mountain in Yasuj, and diagnosing and confirming the type and number of herbarium by a botanist (Herbarium number 91) in Yasuj University, the areal parts were separated and dried in full shade for 2 weeks; we mixed 100 gram of the dried plant with 500 milliliter of 70% ethanol alcohol and took the necessary plant extract in laboratory conditions. The extracts were collected and poured into 500 mg capsules according to standard conditions and stored in the refrigerator's relevant packages.

### 2.3. Compliance with Ethical Standards

The research followed the tents of the Declaration of Helsinki. The Ethics Committee of the Yasuj University of Medical Sciences approved the study (IR.YUMS.REC.1396.1178). All study protocols were approved by the Ethics Committee of the Yasuj University of Medical Sciences. The study also registered as a clinical trial at Iranian Registry of Clinical Trials (IRCT20081011001323N14; https://irct.ir/trial).

### 2.4. Statistical Analysis

All the information were collected and analyzed by using SPSS software version 21. Statistical tests such as the frequency, mean, and standard deviation were used for descriptive data. Then, for analyzing and comparing data between the two groups, the chi-square test and independent *t*-test were used. The significance level was set at 0.05%.

## 3. Results

In the hydrochlorothiazide group, four patients were excluded from the study due to lack of follow-up. The results of the present study showed that before the intervention in terms of mean age, sex, serum urea level (*P*=0.351), serum creatinine (*P*=0.393), stone size (*P*=0178), and the number of stones (*P*=0.052), there was no statistically significant difference between the two treatment groups (Table 1).

Mean serum urea (*P*=0.878), serum creatinine (*P*=0.393), stone size (*P*=0.314), and the number of stones (*P*=0.229) after the intervention in both groups are shown in Table 2. Although the size and number of stones in both groups decreased after intervention, there was no significant difference between them.

In terms of treatment efficacy (stone removal and the presence of residual stones less than 4 mm), although it was above 70% in both groups, no statistically significant difference was observed between both groups (Table 3).

No specific complication was observed in both groups, and about 80% of cases in each group had complete or partial treatment.

## 4. Discussion

The hydroalcholic extract of the areal part of *Alhagi maurorum* plant affects the excretion of kidney stones by an unknown mechanism. It possibly works with a diuretic and spasmolytic effect [7, 8, 10]. Hydrochlorothiazide, which is a sulfonamide derivative, also causes moderate but persistent diuresis of sodium and chlorine. As a result, calcium reabsorption from urine increases, and calcium excretion decreases, resulting in a transient decrease in urinary calcium in absorbed hypercalciuria. For this reason, chronic calcification of calcium stones can be controlled with thiazides [11–14].

This study aimed to compare the effect of the hydroalcholic extract of the areal part of *Alhagi maurorum* plant and hydrochlorothiazide on excretion of 4–10 mm kidney and ureteral stones in patients referred to the urology clinic. The present study results showed that there were no statistically significant differences between the two treatment groups in terms of BUN, Cr, stone size, number of stones, stone location, stone excretion, and the efficacy and side effects of the medicine. In both groups, the number and size of stones decreased significantly after taking medicine, but this decrease was not statistically significant in both groups. Taking these two medicines had no significant effect on BUN and Cr in blood tests. Therefore, to determine the exact effect of these two drugs on these variables, more detailed studies and a higher statistical population are needed.

In a study conducted by Marashdah and Al-Hazimi in 2010, the ethanolic extract (EE) of *Alhagi maurorum* powdered roots was examined for its pharmacological activity. It had dilating and anticontraction properties of the urinary tract and helps expel stones [15]. These properties reported in this study could be a reason for the *Alhagi maurorum* plant's effect on stone excretion in our study.

In another study by Marashdah and Farraj in 2010, one gram of calcium oxalate was added to 5 ml of a 2% solution of the *Alhagi maurorum* extract and mixed for three days. Calcium crystals did not dissolve, and the weight of insoluble calcium oxalate did not change compared to the initial weight [16]. This study shows that the *Alhagi maurorum* plant extract does not reduce the size of calcium crystals and stones, which is not consistent with our study.

A study conducted by Cyrus et al. in 2009 showed that *Alhagi maurorum* extraction increased the excretion of urinary stones by 66% compared to other treatments for colic pain caused by stones. Moreover, it caused a reduction of 2 days in the time required for stone removal, which was not statistically significant [10]. This study's results are a match with our study in terms of the percentage of efficacy of the *Alhagi maurorum* plant. Although in this study *Alhagi maurorum* areal extraction was used instead of whole plant extraction, the effectiveness of this plant on reducing the duration of treatment requires further studies.

A study conducted by Srivastava et al. in 2014 showed that ethyl acetate isolated from the root of *Alhagi maurorum* has an inhibitory effect on urease. The plant methanol extract has a significant diuretic effect and has been effective for a long time for acceptable reduction of acidity and crystallinity. Therefore, it is considered a treatment for urinary tract infections and kidney stones. The extract of this plant has antispasmodic and dilating properties and relieves urinary tract contractions. It significantly removes stones and reduces the time required for urinary stones to pass. However, the extract does not have the property of dissolving calcium stones [17]. Our study had similar results to those of the mentioned study, with the difference in our study that the extract of the *Alhagi maurorum* plant also reduced the size of stones, which may indicate the solubility of this plant.

In a study conducted by Shafaeifar et al. in 2011, there was no statistically significant difference between the numbers of accumulations of calcium oxalate crystals in the studied groups. In this study, it was concluded that the *Alhagi maurorum* plant's aqueous extract is somewhat effective in preventing the formation of calcium oxalate stones in rats by reducing the amount of oxalate and increasing urinary citrate, although it does not affect the amount of calcium oxalate crystals. Due to its analgesic properties and the presence of antioxidant compounds, and the reduction of serum Cr, it may effectively improve pain caused by stones and prevent its effects on kidney tissue [7]. In this study, there was a decrease in the size and number of stones, but more studies are needed to understand the effectiveness of this plant in preventing stone formation and its effect on different types of stones. In addition, in this study, there was no significant difference in Cr levels before and after treatment. It also had little effect on the serum urea content of white and red urine cells, which may partly indicate renal function.

During the study by Mostaanzadeh et al. in 2016, the *Alhagi maurorum* plant extract could prevent the formation of calcium oxalate crystals but had little effect on their dissolution [18].

In a series of randomized studies on thiazide in the treatment of kidney stones, the results were 35% better than in the placebo group. The risks of this treatment if the drug dose is high were 5% [13, 19]. In our study, although complete response in the thiazide group was more, the sum of efficacy was similar in both groups, and in this study, no specific complication was observed in any of the treatment groups.

In another study by Ammar et al, in order to determine the litholytic potential of the extract, calcium oxalate urinary stones were incubated during 12 weeks under continuous shaking in the presence of AME. The results showed a significant dissolution effect of the extract on the kidney calculi during the experimentation period [20]. Although the body status is not similar to the in vitro area, this study also defines the litholytic effect of *Alhagi* and is consistent with our results. The main limitation of this study was confounding factors that interfere in stone passage such as diet, activity, and volume of water, but by randomization, usually it will be similar in both groups.

## 5. Conclusion

The study showed that *Alhagi maurorum* is as effective as hydrochlorothiazide in treatment of kidney and ureteral stones with no significant complications, which is not significantly different in this regard. Moreover, they have no significant effect on renal function tests.

## Figures and Tables

**Table 1:** Demographic characteristics of the patients in the hydrochlorothiazide and *Alhagi* extract groups before treatment.

Treatment group variables	*Alhagi* (intervention)	Hydrochlorothiazide (control)	*P* value
Mean age (years)	45.1 ± 12.77	46.17 ± 14.42	0.73
Sex (female/male)	22/18	23/13	0.29
Mean number of stones	2.45 ± 1.48	2.03 ± 1.54	0.229
Mean size of stones (mil)	13.3 ± 9.163	10.79 ± 6.82	0.178
Mean blood urea (mg/dl)	19.25 ± 2.80	19.61 ± 2.86	0.878
Mean serum creatinine (mg/dl)	0.98 ± 0.18	0.95 ± 0.18	0.393

^*∗*^Independent sample *t*-test was used for analysis.

**Table 2:** Demographic characteristics of patients in both groups after treatment.

Treatment group variables	*Alhagi* (intervention)	Hydrochlorothiazide (control)	*P* value
Mean number of stones	1.33 ± 0.88	0.94 ± 0.79	0.052
Mean size of stones (mil)	6.93 ± 4.15	5.75 ± 5.71	0.314
Mean blood urea (mg/dl)	19.13 ± 2.17	19.61 ± 2.62	0.351
Mean serum creatinine (mg/dl)	0.96 ± 0.18	0.98 ± 0.17	0.588

^*∗*^Independent sample *t*-test was used for analysis.

**Table 3:** Comparison of treatment efficacy in the two studied groups after intervention.

Efficacy	Group/variables		
	Case group		*P* value
	Hydrochlorothiazide	*Alhagi*	
	Numbers (percentage)	Numbers (percentage)	
Complete	14 (38.9%)	8 (20%)	0.305
Partial	14 (38.9%)	24 (60%)	
Unsuccessful	8 (22.2%)	8 (20%)	

^*∗*^Chi-square test was used for analysis.

## Data Availability

The data used to support the findings of this study are available from the corresponding author upon reasonable request.

## Additional Points

*Suggestions*. It is recommended to design a study with a larger sample size, different doses, and longer duration of time, especially in larger kidney stones. It is also recommended to study stones of different sizes and materials.

## References

[1] Tiselius H., Ackermann D., Alken P., Buck C., Conort P., Gallucci M. (2001). Guidelines on urolithiasis1. *European Urology*.

[2] Khan M. S., Lari Q. H., Khan M. A. (2016). Unani concept of nephrolithiasis (hisat-e-kulyah) and its management: an overview. *World Journal of Pharmaceutical and Medical Research*.

[3] Khan S., Thamilselvan S. (2000). Nephrolithiasis: a consequence of renal epithelial cell exposure to oxalate and calcium oxalate crystals. *Molecular Urology*.

[4] Bahmani M., Baharvand-Ahmadi B., Tajeddini P., Rafieian-Kopaei M., Naghdi N. (2016). Identification of medicinal plants for the treatment of kidney and urinary stones. *Journal of Renal Injury Prevention*.

[5] Pearle M. S., Goldfarb D. S., Assimos D. G. (2014). Medical management of kidney stones: AUA guideline. *The Journal of Urology*.

[6] Heilberg I. P., Schor N. (2006). Renal stone disease: causes, evaluation and medical treatment. *Arquivos Brasileiros de Endocrinologia and Metabologia*.

[7] Shafaeifar A. (2012). Effect of hydrophilic extract of Alhagi maurorum on ethylene glycol-induced renal stone in male wistar rats. *Armaghane Danesh*.

[8] Ahmed S., Hasan M. M., Mahmood Z. A. (2017). Antiurolithiatic plants of family Fabaceae: a memoir of mechanism of action, therapeutic spectrum, formulations with doses. *Journal of Pharmacognosy and Phytochemistry*.

[9] Suthar P. (2016). Traditional uses, phytochemistry, pharmacological properties of plant Alhagi maurorum (medik.): A Review.

[10] Cyrus A., Goudarzi D., Jahangiri V. (2010). The effect of Alhagi Pseudalhagi distillate on ureteral stone expulsion. *Journal of Arak University of Medical Sciences*.

[11] Yendt E. R., Cohanim M. (1978). Prevention of calcium stones with thiazides. *Kidney International*.

[12] Choi J. N., Lee J. S., Shin J. I. (2011). Low-dose thiazide diuretics in children with idiopathic renal hypercalciuria. *Acta Paediatrica*.

[13] Yosefi P. (2006). The effectiveness of hydrochlorothiazide on preventing recurrent urinary tract infection in idiopathic hypercalciuric children. *Journal of Arak University of Medical Sciences*.

[14] Vigen R., Weideman R. A., Reilly R. F. (2011). Thiazides diuretics in the treatment of nephrolithiasis: are we using them in an evidence-based fashion?. *International Urology and Nephrology*.

[15] Marashdah M., Al-Hazimi H. (2010). Pharmacological activity of ethanolic extract of Alhagi maurorum roots. *Arabian Journal of Chemistry*.

[16] Marashdah M., Farraj A. (2010). Pharmacological activity of 2% aqueous acetic acid extract of Alhagi maurorum roots. *Journal of Saudi Chemical Society*.

[17] Srivastava B. (2014). Alhagi pseudalhagi: a review of its phyto-chemistry, pharmacology, folklore claims and Ayurvedic studies. *International Journal of Herbal Medicine*.

[18] Mostaanzadeh H., Honarmand E., Khalilian M. (2017). *Chemical Study of The Therapeutic Effect of Alhaji Maurorum Aqueous Extract on Formtion and Dissolution of Calcium Oxalate Kindney Calculi*.

[19] Rezaei Y., Tehranchi A., Mohammadi-Fallah M. (2014). Effects of hydrochlorothiazide on kidney stone therapy with extracorporeal shock wave lithotripsy. *Urology Annals*.

[20] Ammar R. B., Khalifa A., Alamer S. A., Hussain S. G., Hafez A. M., Rajendran P. (2022). Investigation of the potential anti-urolithiatic activity of Alhagi maurorum (Boiss.) grown wild in Al-Ahsa (Eastern Province), Saudi Arabia. *Brazilian Journal of Biology*.

